# Delta shock index as a prognostic marker of perioperative mortality in emergency surgery: A retrospective cohort study

**DOI:** 10.1097/MD.0000000000046975

**Published:** 2026-01-02

**Authors:** Gul Cakmak, Ozlem Atesal

**Affiliations:** a Department of Anesthesiology and Reanimation, Istanbul Training and Research Hospital, Istanbul, Turkey.

**Keywords:** emergency surgery, hemodynamic monitoring, modified shock index, mortality, perioperative risk stratification, shock index

## Abstract

Early identification of high-risk emergency surgical patients is crucial. The shock index (SI) and modified shock index (MSI) are simple vital sign–based parameters with proven prognostic value in trauma, yet their role in non-trauma emergency surgery remains unclear. This study aimed to evaluate the prognostic value of SI, MSI, and their dynamic changes (ΔSI and ΔMSI) between emergency department (ED) admission and the preoperative period in predicting perioperative mortality. This single-center retrospective cohort included 796 adults undergoing emergency surgery within 48 hours of ED admission in 2024. SI and MSI were calculated at ED admission and preoperatively, and ΔSI and ΔMSI were defined as the differences between these time points. The primary outcome was in-hospital mortality; secondary outcomes were postoperative complications. Logistic regression and receiver operating characteristic analyses were used to assess predictive performance. In-hospital mortality was 7.3% (58/796). Non-survivors were older, had higher American Society of Anesthesiologists scores, underwent major surgery more often, and showed higher preoperative heart rate, SI, and MSI with lower blood pressures (*P* < .05). ΔSI predicted mortality with an AUC of 0.73, outperforming ΔMSI (AUC 0.67). A ΔSI ≥ 0.1 showed 87% specificity and was associated with higher mortality (23% vs 4%). In multivariate analysis, ΔSI ≥ 0.1, and both ED and preoperative heart rates remained independent predictors. Dynamic changes in SI between ED admission and preoperative evaluation provide strong prognostic information. A ΔSI threshold ≥ 0.1 is highly specific for mortality risk, supporting its utility as a practical, easily calculated tool for perioperative risk stratification. Prospective multicenter validation is warranted.

## 1. Introduction

The management of emergency surgical patients poses significant challenges, and timely clinical interventions remain a key determinant of survival and postoperative morbidity. Within this context, the role of anesthesiologists is crucial, as responsibilities such as preoperative optimization, intraoperative fluid management, and maintenance of hemodynamic stability directly shape patient outcomes. In perioperative assessment, vital signs provide indispensable indicators because they are readily available and highly reliable. Notably, the shock index (SI) and the modified shock index (MSI), calculated from heart rate and arterial blood pressure, have demonstrated strong associations with intensive care unit admission, postoperative complications, and mortality.^[1,2]^ Therefore, the use of these simple, vital parameter–based scores for early risk stratification in the management of emergency surgery has gained increasing importance.

The shock index is one of the most commonly used parameters in hemodynamic assessment. This simple ratio is calculated as the ratio of heart rate to systolic arterial pressure; an SI value ≥ 1.0 is considered a critical threshold for hemodynamic decompensation.^[3]^ Recent studies have demonstrated that this threshold is strongly associated with mortality, intensive care unit admission, and the need for massive transfusion in trauma patients.^[4,5]^ To further enhance its predictive accuracy, derivative models have subsequently been developed. In particular, the modified shock index, which incorporates mean arterial pressure, has been widely applied to predict mortality in trauma patients and has also emerged as a valuable tool for identifying the risk of hypotension during anesthetic induction in elderly surgical patients.^[6,7]^

The shock index is more sensitive than individual vital signs and can reveal occult hypovolemia even when heart rate and blood pressure remain within normal ranges during the compensatory phase. For this reason, it has been used to detect early hemodynamic deterioration in a variety of critical conditions, including trauma, hemorrhagic shock, pulmonary embolism, myocardial infarction, and sepsis.^[3]^ Growing evidence has emphasized its prognostic value in non-trauma contexts; particularly in patients undergoing major abdominal emergency surgery with high mortality risk, a preoperative SI > 0.9 has been shown to be significantly associated with early mortality, intensive care unit admission, and acute kidney injury.^[1]^ Furthermore, SI has been found to effectively predict postoperative survival in emergency surgical patients admitted to the intensive care unit.^[8]^ Collectively, these findings indicate that both SI and MSI are gaining increasing importance not only in trauma but also in surgical patient management as critical decision-support tools for fluid management and perioperative risk stratification.

The term “delta SI” is used to describe the time-dependent change in the shock index and is most commonly assessed in trauma patients between the prehospital setting and arrival at the emergency department (ED).^[9]^ An increase in SI is considered an indicator of ongoing hemorrhage or suboptimal hemodynamic resuscitation. Several investigations have shown that trauma patients with ΔSI > 0.1 exhibit markedly higher rates of massive transfusion, mortality, and intensive care admission, whereas those with ΔSI ≤ 0.1 demonstrate lower mortality.^[10,11]^

These findings highlight that dynamic alterations in SI constitute a more robust prognostic marker than a single-point measurement. However, the prognostic significance of SI and MSI changes between ED admission and the early preoperative setting remains insufficiently explored in emergency surgical patients. Previous studies have highlighted the importance of SI measurement timing and cutoff standardization for its reliable application, suggesting that SI can be used to identify high-risk patients, prioritize surgical urgency, and guide postoperative recovery pathways.^[10,12]^ Importantly, this perioperative interval constitutes the most critical stage of preoperative evaluation and optimization for anesthesiologists; therefore, assessment of ED–preoperative delta SI and delta MSI may provide valuable insights into the adequacy of fluid resuscitation and overall hemodynamic status, thereby guiding intraoperative decision-making.

This study aimed to evaluate the prognostic significance of the shock index (SI) and the modified shock index (MSI) – measured at both ED admission and in the preoperative period – and, in particular, the predictive value of delta SI and delta MSI changes between these 2 time points as determinants of patient outcomes. Thus, the potential contribution of simple indices based on vital signs to decision-making processes in emergency surgery will be elucidated, and the study also seeks to explore the clinical implications of the ED–preoperative delta SI term, which have been scarcely addressed in the literature

## 2. Material and methods

This investigation was conducted as a single-center retrospective cohort and involved a review of emergency department and operating room records from our institution. The study protocol was approved by the Institutional Review Board (IRB No. 192, July 25, 2025). Owing to its retrospective chart-based design, no additional interventions were undertaken and no direct patient contact occurred for the purpose of data collection. The study was conducted in full compliance with the Declaration of Helsinki.

### 2.1. Patient selection and inclusion criteria

All patients aged 18 years or older who underwent emergency surgical procedures between January and December 2024 were included, provided that surgery was performed within 48 hours of emergency department admission. Exclusion criteria were elective surgery, surgeries outside the study period, incomplete data, pregnancy, and age under 18 years. Accordingly, the final cohort consisted of adult patients undergoing emergency surgery during the defined study period.

### 2.2. Definitions and variables

Demographic characteristics (age and sex), type of surgery, and clinical data such as the American Society of Anesthesiologists (ASA) score were recorded for all patients. In addition, hemodynamic status was evaluated using the shock index and the modified shock index. The SI, originally defined as the ratio of heart rate to systolic blood pressure, serves as a simple indicator of circulatory status, whereas the MSI is calculated as the ratio of heart rate to mean arterial pressure.

These hemodynamic parameters were calculated for each patient at 2 distinct time points: first, at emergency department admission using the initial vital signs, and second, during the preoperative evaluation based on vital signs documented in the anesthesia chart. The differences between these 2 measurements were defined as ΔSI and ΔMSI. Specifically, ΔSI was obtained by subtracting the SI at emergency department admission from the preoperative SI value, whereas ΔMSI was calculated as the difference between preoperative and emergency department MSI values.

The type of surgery was categorized as minor, intermediate, or major based on criteria established in published guidelines and consensus reports to reflect the procedure’s invasiveness, expected duration, blood loss, and need for hospital stay.

Postoperative complications were defined according to standard clinical and guideline-based criteria:

Surgical site or systemic infections: Diagnosed based on clinical signs (fever, erythema, purulent drainage), laboratory findings (elevated white blood cell count, inflammatory markers), and/or positive microbiological cultures, in accordance with Centers for Disease Control and Prevention (CDC) definitions.

Sepsis: Defined according to the Sepsis-3 criteria as life-threatening organ dysfunction caused by a dysregulated host response to infection, identified by a Sequential Organ Failure Assessment score increase of ≥ 2 points.

Reoperation: Any unplanned return to the operating room within the index hospitalization.

Acute kidney injury: Defined according to KDIGO criteria as an increase in serum creatinine by ≥ 0.3 mg/dL within 48 hours, or ≥ 1.5 times baseline within 7 days, or urine output < 0.5 mL/kg/h for at least 6 hours.

Myocardial injury: Diagnosed by elevated cardiac troponin levels above the 99th percentile upper reference limit, with or without ischemic symptoms, as recommended by the Fourth Universal Definition of Myocardial Infarction.

Deep vein thrombosis: Confirmed by duplex ultrasonography in the presence of clinical suspicion (limb swelling, pain, erythema).

Stroke: Defined as an acute neurological deficit of vascular origin persisting for more than 24 hours, confirmed by clinical assessment and neuroimaging.

Prolonged hospitalization: Length of hospital stay exceeding 14 days after surgery.

### 2.3. Outcome measures

The primary outcome was in-hospital mortality, defined as death occurring during hospitalization after surgery. Secondary outcomes included the occurrence of postoperative complications, which were defined as adverse events such as surgical site or systemic infections, sepsis, reoperation, acute kidney injury, myocardial injury, deep vein thrombosis, stroke, prolonged hospitalization, or admission to the intensive care unit. All such complications were systematically recorded.

### 2.4. Statistical analysis

All statistical analyses were performed using IBM SPSS Statistics software (version 25.0; IBM Corp., Armonk). The normality of continuous variables was assessed with the Shapiro–Wilk test. Normally distributed data were expressed as mean ± standard deviation, whereas non-normally distributed variables were summarized as median (interquartile range, IQR). Categorical variables were presented as counts and percentages.

For group comparisons, independent-samples Student’s *t* tests were used for normally distributed continuous variables, while the Mann–Whitney *U* test was applied to non-normally distributed variables. Categorical variables were compared using Pearson’s chi-square test or Fisher’s exact test, as appropriate. When chi-square analyses involved multiple subgroup comparisons (e.g., Table 5), post hoc pairwise tests with Bonferroni correction were applied to control for type I error.

**Table 5:** Comparison of mortality rates between delta shock index (ΔSI) groups.

Mortality	ΔSI < −0.5	−0.5 ≤ ΔSI < −0.1	−0.1 ≤ ΔSI < 0.1	0.1 ≤ ΔSI < 0.5	0.5≤ΔSI	Total
	n (%)					
No	2 (100.0)	88 (96.7)	542 (95.8)	99 (81.1)	7 (46.7)	738 (92.7)
Yes	0 (0.0)	3 (3.3)	24 (4.2)	23 (18.9)	8 (53.3)	58 (7.3)
Total	2 (100)	91 (100)	566 (100)	122 (100)	15 (100)	796 (100)

Pearson’s chi-square analysis was used; χ² (4, N = 796) = 81.31, *P* < .001 was considered statistically significant.

ΔSI = delta shock index.

To identify independent predictors of in-hospital mortality, multivariate logistic regression analysis was performed. Variables with *P* < .05 in univariate analysis were entered into the model, using a forward stepwise selection method. Model performance was further evaluated using Nagelkerke’s *R*^2^, overall classification accuracy.

Receiver operating characteristic (ROC) curve analyses were conducted to assess the discriminative ability of ΔSI and ΔMSI in predicting mortality. The area under the curve (AUC) was calculated, and the optimal cutoff values were determined using the Youden index. To statistically compare the AUCs of ΔSI and ΔMSI, the DeLong test was applied. Diagnostic accuracy measures including sensitivity, specificity, and likelihood ratios were also reported.

A two-tailed *P* value < .05 was considered statistically significant for all analyses.

## 3. Results

The demographic and clinical characteristics of the 796 patients included in the study are presented in Tables 1 and 2. The mean age was 50.5 ± 19.8 years (median: 48.0, range: 18–97), with 66.2% (n = 527) male and 33.8% (n = 269) female. The most frequent ASA classification was ASA II (43.3%, n = 345), while ASA V accounted for 12.7% (n = 101). Regarding surgical procedures, 61.6% (n = 490) were intermediate, 37.8% (n = 301) major, and 0.6% (n = 5) minor.

**Table 1:** Demographic and clinical characteristics of the patients.

Variable	Category	Number (n)	Percentage (%)
ASA score	1	152	19.1
	2	345	43.3
	3	191	24
	4	7	0.9
	5	101	12.7
Gender	Female	269	33.8
	Male	527	66.2
Type of surgery	Major	301	37.8
	Intermediate	490	61.6
	Minor	5	0.6
Postoperative complications (AKI/myocardial injury/DVT/stroke/sepsis/pneumonia)	No	681	85.6
	Yes	115	14.4
Postoperative infection (pneumonia/UTI/SSI)	No	632	79.4
	Yes	164	20.6
Readmission to hospital within 3 months	No	776	97.5
	Yes	20	2.5
Mortality	No	738	92.7
	Yes	58	7.3

AKI = acute kidney injury, ASA = American Society of Anesthesiologists, DVT = deep vein thrombosis, SSI = surgical site infection, UTI = urinary tract infection.

**Table 2:** Descriptive statistics of continuous variables.

Variable	Mean ± SD	Median (Min–Max)
Age	50.49 ± 19.77	48.0 (18.0–97.0)
ED SBP (mm Hg)	139.92 ± 28.5	133.0 (64.0–281.0)
ED DBP (mm Hg)	75.93 ± 15.29	75.0 (40.0–149.0)
ED HR	84.74 ± 17.73	84.0 (0.0–160.0)
ED SI	0.64 ± 0.19	0.63 (0.0–2.34)
ED MSI	1.16 ± 0.33	1.11 (0.0–3.62)
Preop SBP (mm Hg)	137.46 ± 26.62	134.0 (46.0–232.0)
Preop DBP (mm Hg)	78.04 ± 15.34	77.0 (29.0–138.0)
Preop HR	91.08 ± 15.97	90.0 (45.0–158.0)
Preop SI	0.69 ± 0.21	0.67 (0.24–2.39)
Preop MSI	1.22 ± 0.36	1.16 (0.43–3.79)
ΔSI	0.052 ± 0.17	0.049(-0.61–1.99)
ΔMSI	0.059 ± 0.29	0.075(-1.03–2.87)
Hospital LOS (d)	2.32 ± 2.49	1.0 (0.0–17.0)
ICU LOS (ds)	0.95 ± 2.78	0.0 (0.0–18.0)

DBP = diastolic blood pressure, ED = emergency department, HR = heart rate, ICU = intensive care unit, LOS = length of stay, Max = maximum, Min = minimum, MSI = modified shock index, Preop = preoperative, SBP = systolic blood pressure, SD = standard deviation, SI = shock index, ΔMSI = delta modified shock index, ΔSI = delta shock index.

The incidence of postoperative complications was 14.4% (n = 115), with infections observed in 20.6% (n = 164). Re-hospitalization within 3 months occurred in 2.5% (n = 20). The overall in-hospital mortality rate was 7.3% (n = 58) (Table 1).

For continuous variables, mean emergency department (ED) systolic and diastolic blood pressures were 139.9 ± 28.5 and 75.9 ± 15.3 mm Hg, respectively, and mean heart rate was 84.7 ± 17.7 bpm. The average ED SI was 0.64 ± 0.19 (median: 0.63, range: 0–2.34) and ED MSI was 1.16 ± 0.33 (median: 1.11, range: 0–3.62). Preoperatively, the mean systolic and diastolic pressures were 137.5 ± 26.6 mm Hg and 78.0 ± 15.3 mm Hg, while heart rate increased to 91.1 ± 16.0 bpm (median: 90, range: 45–158). The mean preoperative SI was 0.69 ± 0.21 and preoperative MSI was 1.22 ± 0.36. The mean differences were ΔSI = 0.052 ± 0.17 and ΔMSI = 0.059 ± 0.29. Median hospital and ICU stays were 1.0 day (mean 2.32 ± 2.49) and 0.0 days (mean 0.95 ± 2.78), respectively (Table 2).

**Figure 1.**
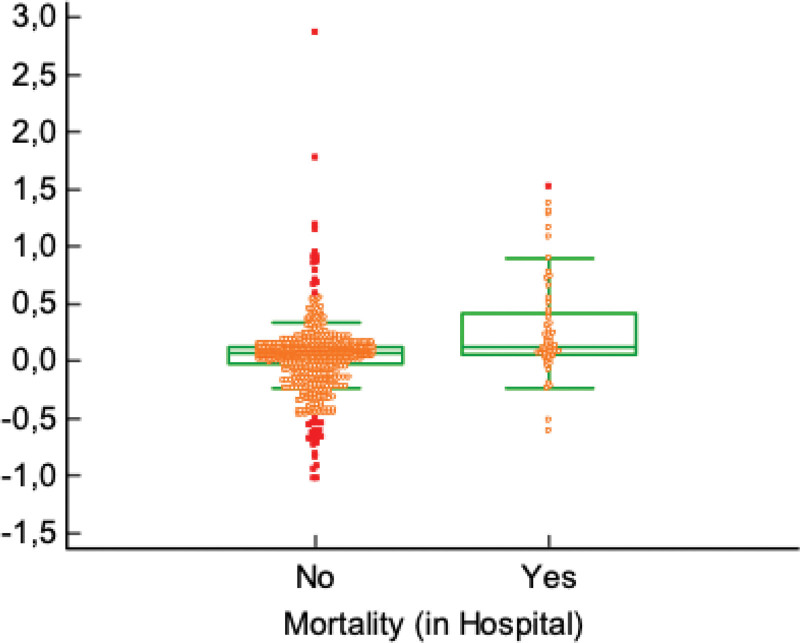
Distribution of shock index according to in-hospital mortality.

Age, ED heart rate, SI, and MSI were all significantly elevated among non-survivors compared with survivors (*P* < .05). Preoperatively, systolic and diastolic blood pressures were significantly lower, while heart rate, SI, and MSI were significantly higher in non-survivors (*P* < .05). ICU stay was also longer in this group (*P* < .001) (Table 3; Fig. 1). ASA classification and type of surgery were significantly associated with mortality (*P* < .001), with higher mortality among ASA V and major surgery patients. Postoperative infections were more frequent among non-survivors than survivors (*P* < .001). No significant differences were observed in terms of sex, overall complications, or re-hospitalization (*P* > .05) (Table 4).

**Table 3:** Comparison of demographic and clinical variables between survivors and non-survivors.

Variable	Mortality		*P* value
	Survivors (n = 738)	Non-survivors (n = 58)	
	Mean ± SD Median (Min–Max)		
Age (years)	49.97 ± 19.59	57.16 ± 21.00	**.008**
ED SBP (mm Hg)	138.42 ± 28.01	131.45 ± 33.26	.073
ED DBP (mm Hg)	75.96 ± 15.14	75.60 ± 17.32	.881
ED HR (bpm)	83.90 ± 16.90	95.48 ± 23.75	**<.001**
ED SI	0.63 ± 0.17	0.79 ± 0.36	**.001**
ED MSI	1.14 ± 0.30	1.34 ± 0.54	**.007**
Preop SBP (mm Hg)	139.10 ± 25.78	116.55 ± 28.52	**<.001**
Preop DBP (mm Hg)	78.48 ± 14.71	72.48 ± 21.25	**.039**
Preop HR (bpm)	89.72 ± 14.86	108.40 ± 19.33	**<.001**
Preop SI	0.67 ± 0.17	1.00 ± 0.37	**<.001**
Preop MSI	1.18 ± 0.31	1.62 ± 0.58	**<.001**
ΔSI	0.047(-0.61–1.99)	0.12(-0.25–1.39)	**<.001**
ΔMSI	0.071(-1.03-2.87)	0.13(-0.61-1.52)	**<.001**
Hospital LOS (d)	1(0-17)	1(0-17)	.142
ICU LOS (d)	0(0-18)	1(0-18)	**<.001**

Independent *t* test and Mann–Whitney *U* test used and *P* < .05 considered significant. Bold values indicate statistical significance at *P* < .05.

DBP = diastolic blood pressure, ED = emergency department, HR = heart rate, ICU = intensive care unit, LOS = length of stay, Max = maximum, Min = minimum, MSI = modified shock index, Preop = preoperative, SBP = systolic blood pressure, SD = standard deviation, SI = shock index, ΔMSI = delta modified shock index, ΔSI = delta shock index.

**Table 4:** Comparison of categorical variables between survivors and non-survivors.

Variable	Mortality		*P* value
	Survivors (n = 738)	Non-survivors (n = 58)	
	N (%)		
ASA score			
ASA 1	146 (19.8)	6 (10.3)	**<.001**
ASA 2	339 (45.9)	6 (10.3)	
ASA 3	170 (23.0)	21 (36.2)*	
ASA 4	6 (0.8)	1 (1.7)	
ASA 5	77 (10.4)	24 (41.4)*	
Gender			
Female	244 (33.1)	25 (43.1)	.120
Male	494 (66.9)	33 (56.9)	
Type of surgery			
Major	255 (34.6)	46 (79.3)*	**<.001**
Intermediate	478 (64.8)	12 (20.7)	
Minor	5 (0.7)	0 (0.0)	
Postop complication			
No	636 (86.2)	45 (77.6)	.073
Yes	102 (13.8)	13 (22.4)	
Postop infection			
No	601 (81.4)	31 (53.4)	**<.001**
Yes	137 (18.6)	27 (46.6)*	
Readmission to hospital within 3 mo			
No	721 (97.7)	55 (94.8)	.179
Yes	17 (2.3)	3 (5.2)	

Pearson’s chi-square analysis used and *P* < .05 considered significant. Bold values indicate statistical significance at *P* < .05.

ASA = American Society of Anesthesiologists, Postop = postoperative.

**Figure 2.**
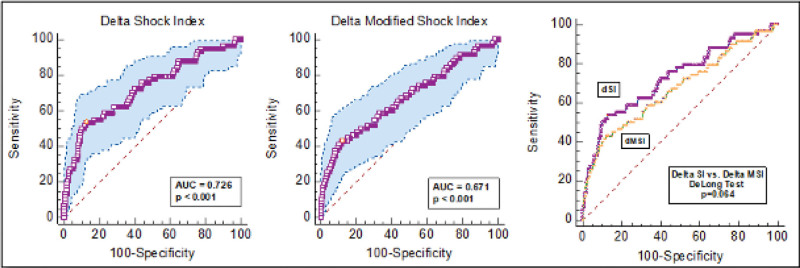
Receiver operating characteristic (ROC) curves for ΔSI and ΔMSI in predicting mortality. Receiver operating characteristic (ROC) curves showing the predictive performance of ΔSI and ΔMSI for in-hospital mortality. AUC values are presented, and comparison between ROC curves was performed using the DeLong test. AUC = area under the curve, ΔMSI = delta modified shock index, ΔSI = delta shock index.

ROC analysis was conducted to assess the diagnostic performance of ΔSI for in-hospital mortality. Among 796 patients (58 deaths, 7.29%), the AUC for ΔSI was 0.73 (95% CI: 0.69–0.76), significantly greater than 0.5 (z = 5.83, *P* < .001) (Fig. 2). The optimal cutoff determined by the Youden index was > 0.106, yielding a sensitivity of 53.5% (95% CI: 39.9–66.7) and a specificity of 87.3% (95% CI: 84.6–89.6). The corresponding positive likelihood ratio was 4.20 (95% CI: 3.09–5.70) and the negative likelihood ratio was 0.53 (95% CI: 0.40–0.70).

ROC analysis for ΔMSI also demonstrated statistically significant discriminatory performance, with an AUC of 0.67 (95% CI: 0.64–0.70; *z* = 4.12, *P* < .001). The optimal cutoff was > 0.1786, corresponding to a sensitivity of 43.1% and specificity of 87.8% (Fig. 2). Although the difference in AUC values between ΔSI (0.73) and ΔMSI (0.67) was 0.055, this was not statistically significant (*P* = .064) (Fig. 2).

Pearson chi-square analysis further revealed significant differences in mortality across ΔSI subgroups, χ² (4, N = 796) = 81.31, *P* < .001. Mortality was 0% in the ΔSI < –0.5 group, 3.3% in–0.5 ≤ ΔSI < –0.1, and 4.2% in–0.1 ≤ ΔSI < 0.1. In contrast, mortality increased to 18.9% in 0.1 ≤ ΔSI < 0.5, and was highest at 53.3% in ΔSI ≥ 0.5 (Table 5).

When patients were categorized as ΔSI < 0.1 (n = 662, 83.2%) or ΔSI ≥ 0.1 (n = 134, 16.8%), no significant differences were found regarding sex (*P* = .797), postoperative infection (*P* = .207), or re-hospitalization (*P* = .824). However, the ΔSI ≥ 0.1 group had significantly higher ASA scores (*P* < .001), with ASA V more frequent (35.1% vs 8.2%), and underwent major surgery more often (54.5% vs 34.4%, *P* < .001). A non-significant trend toward higher postoperative complication rates was observed in this group (19.4% vs 13.4%, *P* = .074). Importantly, in-hospital mortality was significantly greater in the ΔSI ≥ 0.1 group (23.1% vs 4.1%, *P* < .001) Regarding continuous variables, the dSI ≥ 0.1 group had significantly higher emergency department systolic and diastolic blood pressures (*P* < .001 and *P* = .006, respectively), but lower preoperative systolic blood pressure (*P* < .001). They also had a significantly lower heart rate in the emergency department (*P* < .001) but a higher preoperative heart rate (*P* < .001) (Table 6).

**Table 6:** Comparison of demographic, clinical, and outcome variables among ΔSI groups.

Variable	Total (N = 796)	ΔSI < 0.1 (n = 662)	ΔSI ≥ 0.1 (n = 134)	*P* value
	n (%)			
Gender				.797
Female	269 (33.8)	225 (34.0)	44 (32.8)	
Male	527 (66.2)	437 (66.0)	90 (67.2)	
ASA score				**<.001**
1	152 (19.1)	129 (19.5)	23 (17.2)	
2	345 (43.3)	308 (46.5)	37 (27.6)	
3	191 (24.0)	167 (25.2)	24 (17.9)	
4	7 (0.9)	4 (0.6)	3 (2.2)	
5	101 (12.7)	54 (8.2)	47 (35.1)	
Type of surgery				**<.001**
Major	301 (37.8)	228 (34.4)	73 (54.5)	
Intermediate	490 (61.6)	429 (64.8)	61 (45.5)	
Minor	5 (0.6)	5 (0.8)	0 (0.0)	
Postop complications				.074
No	681 (85.6)	573 (86.6)	108 (80.6)	
Yes	115 (14.4)	89 (13.4)	26 (19.4)	
Postop infection				.207
No	632 (79.4)	531 (80.2)	101 (75.4)	
Yes	164 (20.6)	131 (19.8)	33 (24.6)	
Readmission to hospital within 3 mo				.824
No	776 (97.5)	645 (97.4)	131 (97.8)	
Yes	20 (2.5)	17 (2.6)	3 (2.2)	
Mortality				**<.001**
No	738 (92.7)	635 (95.9)	103 (76.9)	
Yes	58 (7.3)	27 (4.1)	31 (23.1)	
	Mean ± SD			
Age	50.5 ± 19.8	50.1 ± 19.7	52.3 ± 20.2	.255
ED SBP	137.9 ± 28.3	136.2 ± 26.3	146.4 ± 36.4	**<.001**
ED DBP	75.9 ± 15.1	75.3 ± 14.4	79.3 ± 18.9	**.006**
ED HR	85.0 ± 17.7	86.2 ± 15.8	77.7 ± 24.0	**<.001**
Preop SBP	138.2 ± 26.5	140.2 ± 25.2	124.0 ± 29.5	**<.001**
Preop DBP	78.1 ± 15.2	78.5 ± 14.8	75.9 ± 17.6	.082
Preop HR	90.7 ± 15.9	89.6 ± 14.9	98.6 ± 18.9	**<.001**
	Median (Min–Max) IQR			
Hospital LOS (d)	1 (0–17) 2	1 (0–17) 2	1 (0–17) 2	.491
ICU LOS (d)	0 (0–18) 0	0 (0–18) 0	0 (0–18) 1	**<.001**

Pearson’s chi square/ Fisher’s exact test used for categorical data, independent *t* test and Mann–Whitney *U* test (for non-parametric distribution) used for continuous data. *P* < .05 considered significant. Bold values indicate statistical significance at *P* < .05.

DBP = diastolic blood pressure, ED = emergency department, HR = heart rate, ICU = intensive care unit, IQR = interquartile range, LOS = length of stay, Max = maximum, Min = minimum, Preop = preoperative, SBP = systolic blood pressure, SD = standard deviation, ΔMSI = delta modified shock index, ΔSI = delta shock index.

Multivariate logistic regression analysis was performed to identify independent predictors of in-hospital mortality. A ΔSI ≥ 0.1, combined with clinical variables, revealed that ED heart rate (OR = 0.890, 95% CI: 0.826–0.959, *P* = .002) and preoperative heart rate (OR = 1.100, 95% CI: 1.010–1.198, *P* = .029) were significant predictors. Preoperative systolic blood pressure showed borderline significance (OR = 0.974, 95% CI: 0.949–1.000, *P* = .054). The explanatory power of the model was moderate (Nagelkerke R² = 0.476), with an overall classification accuracy of 86.2%.

## 4. Discussion

This study demonstrated that changes in the ΔSI between emergency department admission and the preoperative period serve as a strong predictor of in-hospital mortality in emergency surgical patients. The discriminative ability of ΔSI was acceptable, and its high specificity suggests that it is a valid tool for confirming high-risk status in mortality prediction. Clinically most notable, when the ΔSI threshold of ≥ 0.1 was exceeded, the mortality rate increased to 23%, compared with only 4% in those with ΔSI < 0.1. This remarkable contrast emphasizes that even minor increases in ΔSI substantially raise the risk of death by nearly 5-fold, thereby strongly supporting its clinical relevance. In the ROC analysis, the optimal cutoff value for ΔSI was identified as 0.106, and both ΔSI ≥ 0.1 and ΔSI ≥ 0.5 were found to be significantly associated with mortality. However, since a threshold of 0.1 has been more consistently reported as a critical prognostic marker in the literature, the present discussion primarily focused on this cutoff value.^[10–12]^

When examining the clinical profile of non-survivors, several risk factors associated with ΔSI increase were evident. Advanced age, higher ASA scores, a greater proportion of major surgical procedures, and the presence of tachycardia and hypotension in the preoperative period were among the most prominent. This pattern of preoperative hemodynamic instability constitutes a reliable indicator for perioperative complications. Consistent with this, multivariable logistic regression analysis identified heart rate values at both ED admission and in the preoperative period as independent predictors of mortality. In terms of prognostic performance, ΔSI was superior to ΔMSI (AUC: 0.73 vs 0.67). Although this difference was only marginally significant (*P* = .064), the greater ease of calculation and stronger predictive ability of ΔSI support its role as a more practical parameter for clinical application.

Current evidence indicates that the term of ΔSI has been investigated predominantly in trauma populations. In an international trauma study, in-hospital mortality was reported to be approximately 13% among patients with the highest ΔSI values.^[13]^ When compared with these findings, our non-trauma surgical cohort demonstrated a markedly higher mortality rate of 23% at the same threshold (ΔSI ≥ 0.1). This discrepancy is likely attributable to the fact that emergency surgical patients are generally older and burdened with more comorbidities, thereby underscoring the prognostic significance of ΔSI increase in this population.

Prior research in elderly populations provides further validation for this concept. In particular, Funabiki et al^[12]^ demonstrated that among trauma patients aged over 65 years, a ΔSI > 0.1 was strongly associated not only with higher mortality but also with an increased need for emergent hemostatic surgery. This finding is particularly relevant to perioperative practice, as it is well recognized that conventional vital sign changes are often attenuated in older adults. Within this context, ΔSI emerges as a more sensitive and clinically valuable parameter for informing early decision-making in this vulnerable cohort.

Trauma research has consistently demonstrated the prognostic significance of a ΔSI threshold greater than 0.1. Joseph et al^[13]^ reported that even in trauma patients despite initial hemodynamic stability, a ΔSI > 0.1 in the emergency department was associated with an increased risk of mortality, a higher frequency of emergent exploratory laparotomy, and greater in-hospital complication rates. These findings convey an important implication for anesthetic practice: even patients presenting with clinical stability may require close hemodynamic monitoring and aggressive fluid resuscitation when ΔSI increase is present.

Evidence from large-scale cohort analyses underscores the clinical relevance of this threshold. For instance, Walker et al^[14]^ reported that trauma patients with ΔSI > 0.1 more frequently required massive transfusion and had a substantially increased risk of mortality compared with those with lower values. Similarly, Wu et al^[15]^ demonstrated that ΔSI > 0.1 was strongly associated with increased ICU admission and in-hospital complications. In our study, the specificity of ΔSI ≥ 0.1 was approximately 87%, consistent with these findings and underscoring its strength in reliably identifying high-risk patients. This high specificity further implies a low false-positive rate, thereby minimizing unnecessary interventions in clinical practice.

Walker et al^[14]^ also demonstrated that ΔSI improved prognostic accuracy when integrated with other clinical parameters. In contrast, their analysis was limited to patients with severe trauma (ISS ≥ 9), whereas our study included all emergency surgical patients classified as ASA physical status I or higher. This broader and more heterogeneous cohort strengthens the clinical applicability of our findings to routine anesthetic practice, which typically involves a more diverse patient population.

The prognostic value of ΔSI has been shown to depend on the interval between measurement points, underscoring the importance of standardizing the timing of assessments across different clinical settings. Bardes et al^[16]^ demonstrated in a rural trauma population that a ΔSI > 0.1 represented a critical threshold predictive of transfusion requirements and ICU admission, and further suggested that transport time could influence this relationship. In our study, the lack of a detailed analysis regarding the interval from emergency admission to surgery constitutes a limitation, yet it also underscores an important parameter that warrants investigation in future research. Notably, the AUC of 0.73 observed in our univariate model suggests that, due to the characteristics of our cohort, the discriminative capacity of ΔSI may be slightly higher than that reported in the trauma literature.

Dynamic alterations in the shock index are gaining prominence as early warning signals of poor prognosis, extending their clinical utility well beyond the trauma setting. Huang et al^[17]^ demonstrated that in critically ill ICU patients, a positive ΔSI during the emergency department stay was significantly associated with increased in-hospital mortality, with the effect being particularly pronounced among elderly individuals and those with sepsis. Consistent with these findings, our analysis also identified ΔSI increase as a strong predictor of mortality in emergency surgical patients. Interestingly, neither Huang’s cohort nor our own revealed a significant association between ΔSI and hospital length of stay, suggesting that while ΔSI effectively reflects acute deterioration and early mortality risk, additional parameters may be required to predict long-term outcomes.

The risk factors identified in previous studies align closely with the findings of our investigation. Liao et al^[6]^ demonstrated that both the Shock index (SI) and the modified shock index (MSI) are reliable predictors of mortality, transfusion requirements, the need for emergent surgery, and intensive care unit admission in trauma populations. Similarly, Joseph et al^[13]^ reported that even patients presenting with initially stable vital signs had worse outcomes at a ΔSI greater than 0.1. In patients with gastrointestinal hemorrhage, Loewe et al^[18]^ demonstrated that SI measured at emergency department admission was predictive of mortality; however, Golcuk et al^[19]^ suggested that ΔSI could provide stronger prognostic value compared with a single baseline measurement. This finding suggests that parameters reflecting dynamic hemodynamic changes (e.g., ΔSI) may be more reliable than static measurements. In our study, although other vital parameters (heart rate, blood pressure, MSI) were also associated with mortality, multivariable analyses identified ΔSI as an independent and stronger predictor, highlighting the importance of integrating dynamic changes into clinical decision-making models.

Our findings strongly support ΔSI as a reliable prognostic indicator. In a substantial proportion of patients, mortality risk was effectively identified on the basis of ΔSI values. In terms of perioperative risk assessment, the significantly higher mortality observed in patients with ΔSI ≥ 0.1 indicates that this threshold represents a clinically relevant cutoff, emphasizing the necessity of tailoring perioperative management accordingly.

However, it should be acknowledged that, in clinical practice, certain high-risk patients can still present with ΔSI values below 0.1. Therefore, a comprehensive approach is warranted: dynamic changes in SI should be interpreted in conjunction with baseline vital signs. Indeed, our logistic regression analysis revealed that both blood pressure and heart rate values recorded in the emergency department and the preoperative period were also identified as independent predictors of mortality. This finding has practical implications for anesthetic practice: while ΔSI calculation can be incorporated into routine preoperative assessment, it should serve as a complementary tool rather than a replacement for conventional vital sign monitoring.

In this study, the change in the modified shock index (ΔMSI) was also comprehensively evaluated in terms of prognostic significance. Our results demonstrated that ΔMSI showed lower predictive performance for mortality compared with ΔSI (AUC = 0.67 vs 0.73). Although this difference did not reach conventional statistical significance (*P* = .064), the relative simplicity of calculation and the higher discriminative ability of ΔSI support its potential role as a more practical indicator in clinical settings.

The paucity of studies directly evaluating ΔMSI highlights the existing knowledge gap in this field. Current evidence has largely focused comparing the conventional SI, ΔSI, and other variations of the shock index. A comprehensive trauma analysis by Asim et al demonstrated that mortality occurred at a markedly higher rate among patients with ΔSI > 0.1, and that elevated ΔSI predicted mortality with 77% specificity and a 99% negative predictive value.

Our study demonstrates that dynamic changes in the shock index (ΔSI), by reflecting time-dependent hemodynamic fluctuations, provide a stronger and more practical parameter compared with ΔMSI. Moreover, our findings underscore the paucity of evidence regarding ΔMSI in the literature and highlight the need for future comparative studies in this field. From an anesthetic practice perspective, the combination of calculation simplicity and higher predictive value supports the utility of ΔSI as a practical tool for routine clinical application.

### 4.1. Study limitations

The retrospective design limits the ability to draw causal inference and introduces a potential risk of selection bias. The external validity of the findings to institutions with heterogeneous patient populations and varied clinical settings may be restricted. In addition, the timing of ΔSI measurements lacked methodological consistency, and the influence of the interval between emergency department admission and the preoperative period was insufficiently investigated. The relatively few patients within the high ΔSI subgroups restrict the strength of inferences, while the inability to incorporate all potential confounders into the model further limits the interpretation of results. Therefore, prospective studies are warranted to validate and expand these findings.

Future research directions include multicenter prospective validation studies assessing the applicability of ΔSI among heterogeneous populations and practice settings, real-time implementation of ΔSI-based early warning systems. Furthermore, machine learning algorithms that integrate ΔSI with other clinical parameters, as well as cost-effectiveness analyses examining the health economic implications of ΔSI-based risk stratification, may provide valuable contributions. The development of technological infrastructure for real-time perioperative monitoring of ΔSI and its integration into standardized protocols constitutes a key research priority.

## 5. Conclusion

This study demonstrates that ΔSI is a valuable and practical parameter for predicting mortality in emergency surgical patients. Consistent with findings from the trauma literature, a ΔSI threshold of ≥ 0.1 emerges as a critical indicator of risk. Nevertheless, the incorporation of this parameter into routine clinical practice will require prospective validation studies and the development of standardized protocols

ΔSI has the potential to complement existing risk assessment tools and contribute to early risk stratification in emergency surgical populations. Its high specificity (87%) and ease of calculation represent strong arguments in favor of its integration into routine clinical practice. Future prospective investigations will be crucial to further elucidate the capacity of ΔSI to enhance perioperative care quality and potentially inform the establishment of new standards in anesthetic practice.

## Author contributions

**Conceptualization:** Gul Cakmak.

**Data curation:** Gul Cakmak, Ozlem Atesal.

**Formal analysis:** Gul Cakmak.

**Funding acquisition:** Gul Cakmak, Ozlem Atesal.

**Methodology:** Gul Cakmak, Ozlem Atesal.

**Project administration:** Gul Cakmak.

**Resources:** Gul Cakmak, Ozlem Atesal.

**Software:** Gul Cakmak, Ozlem Atesal.

**Supervision:** Gul Cakmak.

**Validation:** Ozlem Atesal.

**Visualization:** Gul Cakmak.

**Writing – original draft:** Gul Cakmak.

**Writing – review & editing:** Gul Cakmak.
